# Evolution of the intrinsic electronic phase separation in La_0.6_Er_0.1_Sr_0.3_MnO_3_ perovskite

**DOI:** 10.1038/s41598-016-0009-0

**Published:** 2016-12-05

**Authors:** Lili Chen, Jiyu Fan, Wei Tong, Dazhi Hu, Yanda Ji, Jindong Liu, Lei Zhang, Li Pi, Yuheng Zhang, Hao Yang

**Affiliations:** 1 Department of Applied Physics, Nanjing University of Aeronautics and Astronautics, Nanjing, 210016 China; 2 High Magnetic Field Laboratory, Chinese Academy of Sciences, Hefei, 230031 China

## Abstract

Magnetic and electronic transport properties of perovskite manganite La_0.6_Er_0.1_Sr_0.3_MnO_3_ have been thoroughly examined through the measurements of magnetization, electron paramagnetic resonance(EPR), and resistivity. It was found that the substitution of Er^3+^ for La^3+^ ions introduced the chemical disorder and additional strain in this sample. An extra resonance signal occurred in EPR spectra at high temperatures well above T_*C*_ gives a strong evidence of electronic phase separation(EPS). The analysis of resistivity enable us to identify the polaronic transport mechanism in the paramagnetic region. At low temperature, a new ferromagnetic interaction generates in the microdomains of Er^3+^-disorder causing the second increase of magnetization. However, the new ferromagnetic interaction does not improve but decreases electronic transport due to the enhancement of interface resistance among neighboring domains. In view of a really wide temperature region for the EPS existence, this sample provides an ideal platform to uncover the evolution law of different magnetic structures in perovskite manganites.

## Introduction

Perovskite manganites with a general formula of R_1−*x*_A_*x*_MnO_3_ (where R is a trivalent rare-earth element such as La, Pr, Sm, and A is a divalent alkaline-earth element such as Ca, Sr, and Ba) have received much attention due to their unusual electron-transport and magnetic properties, which are indispensable for applications in microelectronic, magnetic, and spintronic devices^1–3^. These fascinating properties mainly include metal-insulator transition (MIT), colossal magnetoresistance, and magneto-caloric effect, which are due to strong coupling between the spin, charge and orbital ordering^4–6^. Correlation between them exhibits a significant changes in magnetic transitions such as transformation from paramagnetic (PM) to ferromagnetic (FM) transition and FM to antiferromagnetic (AFM) transition. The coexistence and competition between the double exchange (DE) and super-exchange interaction is related to FM and AFM properties in manganites, respectively^7,8^.

Normally, in a fully homogeneous DE system, one would expect a sharp MIT from the low temperature metallic FM phase into the high-temperature insulating PM phase at the Curie temperature T_*C*_^9,10^. However, controversial cases usually appeared in some actual materials. Sometime, the temperature of MIT is higher than T_*C*_ or a metallic (insulating) state occurs in PM (FM) phases, which can not be completely understood only by DE mechanism^11–13^. There is now compelling experimental evidence that the complex interaction of the freedom leads to a electronic phase separation (EPS) instead of a single homogeneous phase. For example, in the protoptypical manganite (La_1−*y*_Pr_*y*_)_1−*x*_Ca_*x*_MnO_3_, the competition among the ferromagnetic metallic, charge ordered insulating and paramagnetic insulating phases brings multiphase coexistence over a board range of temperatures^14,15^. In fact, except for perovskite manganite, other correlated electronic materials such as high-T_*C*_ superconductor and multiferroicity also show a similar EPS behavior. Generally, EPS is categorized as extrinsic or intrinsic according to its origin. The extrinsic EPS stems from external perturbation such as the strain applied by the substrate in film sample, whereas the intrinsic EPS is generated by the indigenous property of materials which are inclined to be inhomogeneous^5,16,17^. Up to now, some inspections have proved that the shape and scale of EPS in manganites are different for variational systems with domain sizes ranging from a few nanometers to several micrometers^18–21^. According to the length scale of EPS, different techniques are carried out to detect the signatures of EPS. Generally, for the nanoscopic EPS in manganites, both high-resolution transmission electron microscopy and scanning tunneling spectroscopy can be used to reveal the coexistence of nanoscopic charge-ordered (AFM insulating) domains and the FM metallic domains, giving the local structural information at atomic level^16,22^. For mesoscopic phase separation, diffraction techniques can be used to reveal its distinct features since the size scale of the inhomogeneities is large enough to produce well-defined reflections in neutron and X-ray diffraction patterns^23,24^.

Electron paramagnetic resonance (EPR) is known as a powerful probe of spin dynamics and magnetic correlation in magnetic materials on a microscopic level. The signal of EPR is sensitive to the variation of localized environment of magnetic ions. Due to this feature, EPR has been also applied to identify the existence of EPS in some manganites^25,26^. EPR of Mn^3+^ ions may provide useful information about the short-range ordering of the paramagnetic ions since that EPR absorption spectra show distinct resonance lines for the ions involved in structural units of well-defined symmetry and those connected in clusters^27,28^. In this article we report a striking intrinsic EPS in the lightly doped La_0.6_Er_0.1_Sr_0.3_MnO_3_ (LESMO) probing by the EPR technology and magnetization measurements. It was found that the LESMO sample generated a PM-FM phase transition at the Curie temperature T_*C*_ = 300 K. However, The coexistence of PM phase and FM microdomain/clusters was identified at 330 K, well above T_*C*_. Moreover, we can also observe an obvious MIT from the measurement of electronic transport at high temperature 325 K. All evidences reveal that an EPS behavior exists in the LESMO sample at 300 K ≤ T ≤ 330 K. We suggest that the substitution of Er^3+^ ions which induce the chemical disorder and the crystal distortion are main reasons for the observed EPS in LESMO sample.

## Results and Discussion

**Figure 1:**
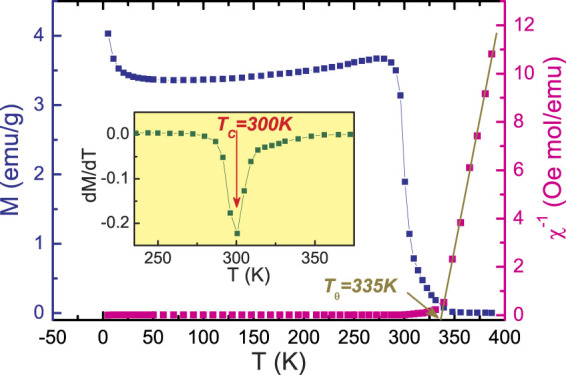
Left axes: Temperature dependence of magnetization measured at H = 100 Oe for La_0.6_Er_0.1_Sr_0.3_MnO_3_. Right axes: Inverse susceptibility *χ* ^−1^ dependence of temperature and the solid line represents the fitting data according to the Curie-Weiss law. Inset shows the plots of dM/dT versus T.

Figure 1(left-hand axis) shows the magnetization (M) versus the temperature (T) plot in H = 100 Oe magnetic field, which depicts a very sharp PM-FM phase transition. The observed minimum in the dM/dT versus T plot identifies the Curie temperature (T_*C*_ ∼ 300 K) of the sample(inset of Fig. 1). To get a clear knowledge about the magnetic interaction, the inverse susceptibility 1/*χ* versus temperature has been also plotted in Fig. 1(right-hand axis). For a ferromagnet, it is well known that in the PM region, the relation between *χ* and T should follow the Curie-Weiss law, i.e. $\chi =\frac{C}{T-{T}_{\theta }}$, where T_*θ*_ is the Curie-Weiss temperature and C is the Curie constant defined as: $C=\frac{{N}_{A}}{3{k}_{B}}{({\mu }_{eff}^{exp})}^{2}$, N_*A*_ = 6.023 × 10^23 ^mol^−1^ is the number of Avogadro, k_*B*_ = 1.38016 × 10^−16^ erg K^−1^ is Boltzmanns constant, and ${\mu }_{eff}^{exp}$ is the experimental effective moment. A linear fit to high temperature yields Curie constant (C = 4.68 emu K/mole Oe) and positive Curie-Weiss temperature T_*θ*_ = 335 K. Obviously, the T_*θ*_ is much higher than T_*C*_. This discrepancy may be associated to the presence of FM clusters above T_*C*_^29^. The theoretical paramagnetic effective moment(${\mu }_{eff}^{the}$) of LESMO sample should be given by the relation^30^:

1 $${\mu }_{eff}^{th{e}^{2}}=0.1{g}_{E{r}^{3+}}^{2}{J}_{E{r}^{3+}}({J}_{E{r}^{3+}}+\mathrm{1)}+0.7{g}_{M{n}^{3+}}^{2}{J}_{M{n}^{3+}}({J}_{M{n}^{3+}}+\mathrm{1)}+0.3{g}_{M{n}^{4+}}^{2}{J}_{M{n}^{4+}}({J}_{M{n}^{4+}}+\mathrm{1)}$$

where g is the Lande factor and J = (L + S) is the total angular moment. For Mn^3+^/Mn^4+^ ions, we have J = S due to the absence of orbital moment. It can be deduced that the theoretical paramagnetic effective moment ${\mu }_{eff}^{the}$ is equal to 5.52 *μ*_*B*_, which is slightly smaller than the experimental effective moment ${\mu }_{eff}^{exp}$ of 6.12 *μ*_*B*_ (${\mu }_{eff}^{exp}=2.83\sqrt{C}{\mu }_{B}$). The discrepancy between the experimental and the theoretical values implies that partial short-range FM couplings might have been developed in the PM region contributing to the additional magnetic moments. This phenomenon is generally termed as eletronic phase separation and has been extensively reported in other manganites^31,32^.

**Figure 2:**
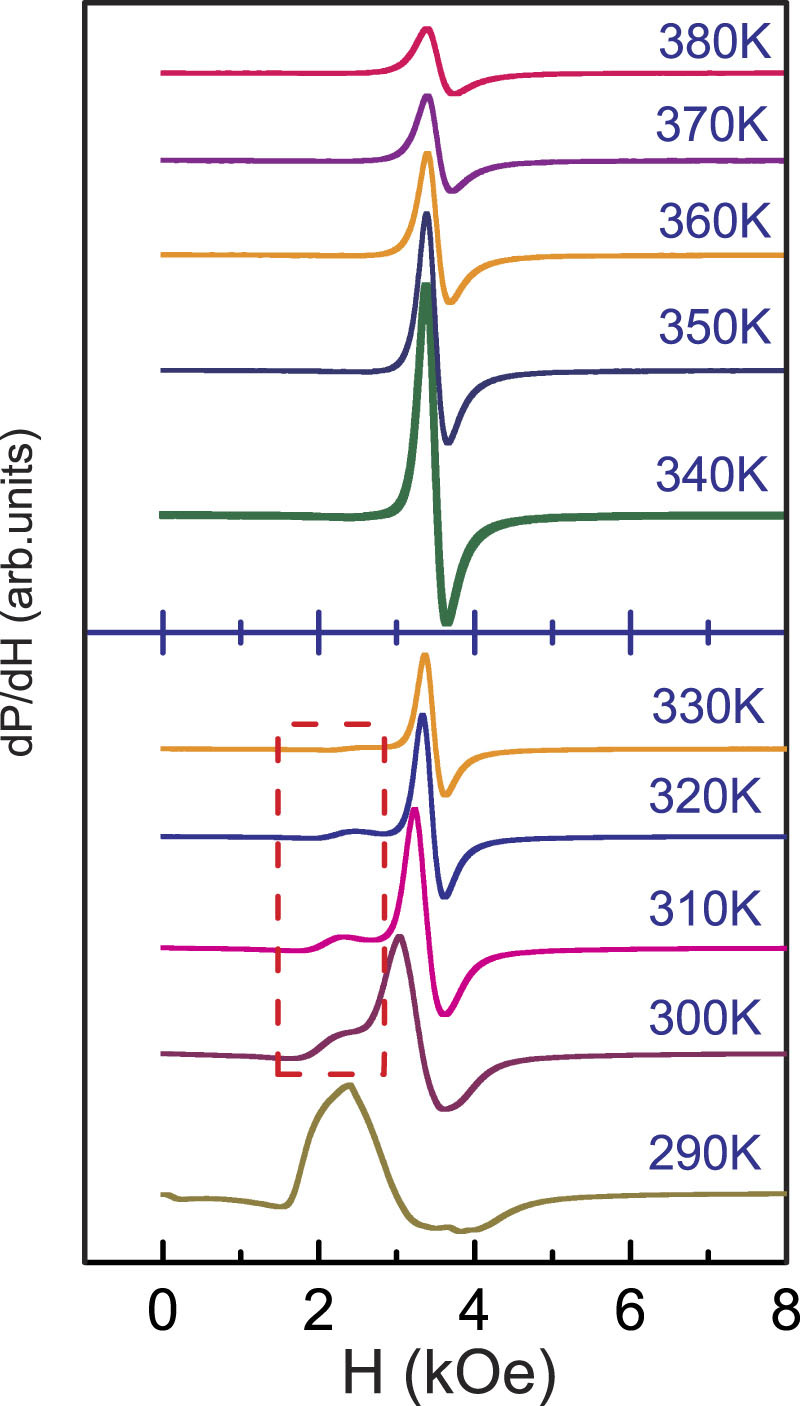
EPR spectrum of manganite La_0.6_Er_0.1_Sr_0.3_MnO_3_ at temperatures of 290 K ≤ T ≤ 380 K.

To further identify the presence of EPS, we performed the EPR measurement on the LESMO sample. Many researches about spin-lattice coupling and spin-spin relaxation on bulk manganites have been reported by means of EPR^33–37^. Not only that, EPR is an important tool to probe EPS in perovskite manganites^26^. Deisenhofer *et al.* reported the directed observation of FM clusters in paramagnetic La_0.875_Sr_0.125_MnO_3_ single crystals by using EPR^38^. As shown in Fig. 2, a series of EPR resonance spectra (*dp/dH*) from 380 to 290 K across the phase transition regime are measured. At T ≥ 340 K, a series of single PM resonance line are shown in the upper part of Fig. 2. The appearance of single PM resonance lines just corresponds to the paramagnetic state in M(*T*) curve of Fig. 1. Blow 340 K, however, besides the initial PM resonance spectra which basically do not show any shifting, a nascent resonance peak is visible at the low field region of ∼2500 Oe. Comparing with PM resonance lines, the extra peak shows a notable difference which is strongly dependent on the change of temperature. As shown in lower part of Fig. 2, with the decrease of temperature, the intensity of this peak not only increases but also the peak position gradually moves towards lower field region. In some manganites with EPS, this low field peak has been referred to the fingerprint of FM microdomain or clusters^39^. In fact, combining the previous finding in Fig. 1 where the Curie-Weiss temperature is higher than 30 K different from the Curie temperature, we can realize that some localized FM interactions have generated in PM state. Therefore, from 330 to 300 K, two peaks coexist in the same temperature indicating that two different magnetic phases coexist in the system. Until to T = 290 K, the renascent resonance peak together with the initial PM peak evolves into a single broad peak, which further shifts to lower field region. In Fig. 1, at T < 300 K, one can find that the sample has fulfilled PM-FM phase transition and all PM phases completely turn to FM phases. Consequently, at T = 290 K, there is only a single FM resonance peak to be measured. Obviously, the variations of EPR are in excellent agreement with the magnetization of Fig. 1, giving a further confirmation for the presence of EPS in LESMO sample.

At present, EPS phenomenon has been received more and more attention since that a mass of strong experimental evidences indicate that EPS is strongly correlated with the magnetic and electron transport properties of perovskite oxide manganites^40,41^. For instance, the earlier speculation that the presence of colossal magnetoresistance behavior is the result of emergent EPS has been widely confirmed^18,42^. However, the origin and formation of EPS are still debated. From the view of theoretical research, five different models were suggested to analyze the EPS mechanism. (1) one-orbital FM Kondo model^43^; (2) two-orbital model with Jahn-Teller phonons^44^; (3) a model Hamiltonian including electron-phonon interactions and long-range elastic coupling^15^; (4) a microscopic model includes all the key energy scales^45^; (5) a phenomenological model using Ginzburg-Landau theory approach^46^. Each model can partially explain the EPS behavior in some specific systems. On the other hand, from the view of experimental research, the local chemical disorder and complex electroic/magnetic interaction are the key mechanisms for the presence of EPS^47,48^. In addition, inherent crystalline strain and local lattice distortions have been also identified to have a strong influence over the formation of EPS. Here, all of these factors are possibly responsible for the observed EPS in the present LESMO sample. For the pristine La_0.7_Sr_0.3_MnO_3_, it has no EPS behavior and shows pure PM phase above T_*C*_ = 370 K. With the substitution of Er^3+^ ions, the extent of crystal mismatch is increased due to the smaller ionic radius of Er^3+^ than La^3+^ (${{\rm{R}}}_{E{r}^{3+}}{/}_{L{a}^{3+}}$ = 1.062/1.216 Å), giving rise to more strain among crystal lattices. Obviously, the larger the crystal mismatch, the larger the crystal distortion and the strain. Meanwhile, the Er^3+^ doping necessarily generates a certain kind of chemical disorder because the spatial locations of the doping ions are random. Some areas maybe gather more Er^3+^ ions while the other areas gather more La^3+^ ions. They are scattered in the system. Different regions generate variational magnetic domains and show different phase transitions. When some parts start to forme FM coupling, others remain a PM state. Consequently, the system exhibits an EPS behavior.

**Figure 3:**
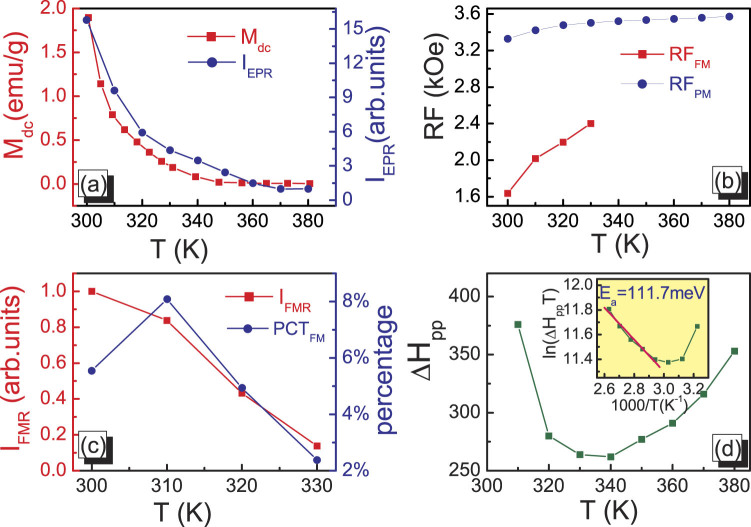
(**a**) EPR intensity I_*EPR*_ versus T (solid circles) and M_*dc*_ versus T (solid squares) at PM region. (**b**)Resonance field (RF) dependence of temperatures for PM and FM peak. (**c**) FM intensity (I_*FMR*_) and FM fraction percentage (PCT_*FM*_) dependence of temperatures. (**d**) EPR linewidth Δ*H*_*PP*_ versus *T*; inset represents the EPR linewidth plotted as Ln(Δ*H*_*PP*_T) versus 1000/T, (the solid line represents the fitting result with Eq. (2)).

For further understanding the EPS nature and features in this sample, some relevant parameters including resonance intensity, resonance field position and resonance peak width are shown in Fig. 3. The intensity of EPR spectra is an important parameter to identify the magnetic ion contribution to the resonant entities. Therefore, the variation of EPR intensity should be basically consistent with the change of magnetization of Fig. 1. Here, we used the I_*EPR*_ to denote PM resonance intensity which can be calculated directly by double integral for EPR curves of Fig. 2. The variation of I_*EPR*_ from 380 to 300 K crossing the whole EPS and pure PM regime are shown in Fig. 3(a, right-hand axis). For comparison, the left-hand axis of Fig. 3(a) shows the variation of magnetization at the same temperature range. Obviously, the change of I_*EPR*_ basically agrees with the variation of M(T), indicating that the magnetic variations detected from EPR spectra are in perfect accordance with the observation from magnetization measurements. The resonance field position (RF) is another key parameter whose behavior can clearly show the variation of magnetic phase. Two curves in Fig. 3(b) depict the RF dependence of temperatures for PM and FM peak, respectively. One can find that the position of PM peak is almost around the magnetic field of H = 3500 KOe showing a weak temperature dependence. On the contrary, the FM peak fleetly shifts to low field region with the decrease of temperature, implying that the size and FM coupling intensity of microdomain constantly grow in EPS region. In order to uncover the detailed evolution of FM microdomain in EPS region, we adopted a proportion parameter (PCT_*FM*_) to describe the development of FM microdomain in PM background. The percentage PCT_*FM*_ is defined as I_*FMR*_/(I_*FMR*_ + I_*EPR*_). Meanwhile, the numerator (I_*FMR*_ is defined as the resonance intensity of FM microdomain and deduced by the double integral for the low field resonance spectra in Fig. 2.) only contains the contribution from FM microdomain. As shown in Fig. 3(c), the value of I_*FMR*_ exhibits a sustained growth with the decrease of temperatures indicating the development and ordering of FM microdomain in EPS region. However, the right-hand axis shows that the PCT_*FM*_ does not keep a sustained growing but turns to decrease at T < 310 K. Near the Curie temperature 300 K, most of “dormant PM” regions start to be active and promptly form a global FM phase transition. Thus, at T = 300 K, the PCT_*FM*_ is naturally reduced. Figure 3(d) shows the temperature dependence of the EPR line width ΔH_*PP*_, which is defined as the width between the highest and the lowest points in the EPR spectrum. In the EPR research on perovskite manganites, the broadening of linewidth is an usual phenomenon. Generally, when the temperature approaches T_*C*_ from above and departs from T_*C*_, the EPR linewidth always first decreases and then increases^49–51^. Therefore, a minimum linewidth occurs around T_*C*_. Here, the minimum linewidth occurs at T_*min*_ = 340 K, far above the Curie temperature 300 K. It once again testifies the presence of the magnetic inhomogeneity at T_*C*_ < T < T_*min*_, consistent with the EPS observed in this temperature region.

So far, for the most of hole-doped manganites, the conduction mechanism is generally considered by the adiabatic small polaron hopping model in PM region. In the earlier research, one similar behavior between temperature dependence the EPR linewidth and conductivity has been also found^52^. Shengelaya *et al.* proposed a “bottleneck model” to describe the Δ*H*_*PP*_ vursus temperature by using the following equation^49^:

2 $${\rm{\Delta }}{H}_{PP}(T)={\rm{\Delta }}{H}_{0}+\frac{A}{T}exp(-{E}_{a}/{k}_{B}T)$$

where A is a constant and E_*a*_ is the activation energy. Meanwhile, the activation energy E_*a*_ can be obtained from fitting data by using the above expression. The inset of Fig. 3(d) plots the ln(Δ*H*_*PP*_ *T*) versus 1000/T and the straight line represents the fitting results. the activation energy E_*a*_ is 111.7 meV. As for hole-doped manganites, the conductance is achieved by the e_*g*_ electronic hoping. Due to lattice distortion, the mobility of e_*g*_ electron is reduced resulting in the localization of carriers and the formation of polaron. Here, the substitution of Er^3+^ ion on A-site sublattice exacerbates lattice distortion giving rise to a large activation energy. Therefore, the high activation energy implies a strong electron-lattice coupling in the LESMO sample and the carriers are polarons.

**Figure 4:**
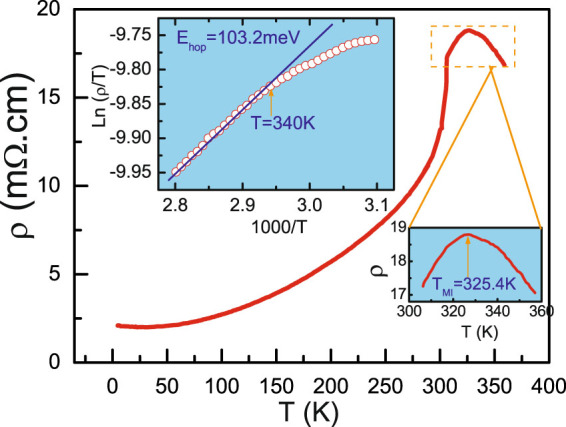
The resistivity as a function of temperature for La_0.6_Er_0.1_Sr_0.3_MnO_3_. The left inset shows Ln(*ρ*/T) versus 1000/T and the solid line is the linear fit according to Eq. (3) and the right one shows the magnified *ρ*(T) in the EPS region.

The variation of electrical resistivity with temperature of the LESMO sample is shown in Fig. 4. It can be seen that the conduction behavior presents a noticeable MIT at 325.4 K. Below 325.4 K, the resistivity decreases monotonically with decreasing temperature. Obviously, at 300–325 K, the system is at PM metallic state. To confirm the nature of hopping conduction and to observe the strength of the electron-phonon interaction in PM phase, as shown in the left inset of Fig. 4, we have fitted temperature dependent resistivity data using small polaron hopping model. The expression of electrical resistivity is given by

3 $$\rho (T)={\rho }_{0}Texp(\frac{{E}_{hop}}{{k}_{B}T})$$

where E_*hop*_ denotes as hopping energy of polarons^53–55^. According to the theoretical descriptions^56^, the small polarons were caused by strong electron-phonon coupling and the hopping energy E_*hop*_ responses to localization and de-localization of the carriers in transport properties. The small polaron expression is represented a linear line in a ln$(\frac{\rho }{T})$ versus $\frac{1}{T}$ plot and the hopping energy E_*hop*_ is fitted to be 103.2 meV, which is very close to the activation energy 111.7 meV deduced from the EPR linewidth. Here, the magnetic polaron model can be used to understand the observed MIT at 325.4 K. The magnetic polaron forms by self-trapping in a ferromagnetic cluster of spin below the T_*p*_ (T_*p*_ denotes the temperature of polaron formation). As the temperature decreases towards T_*C*_ from above, the magnetic polarons grow in size. Our results of Fig. 3(c) just verify this variation. Majumdar and Littlewood (ML) ever suggested that^57^, for a magnetic insulating material with low carrier density, some separated polarons could overlap for each other as the condition of n*ξ* ≈ 1 was satisfied (n and *ξ* denote the carrier density and ferromagnetic correlation length, respectively.). Once the polarons overlap, the carriers will be delocalized and the polarons become free carriers, which instantaneously causes a rapid increase of electronic conductivity. In the present LESMO system, the substitution of Er^3+^ ions causes the crystal distortion, which brings two effects: (1) It decreases the Mn-O-Mn bond angle and suppresses the e_*g*_ electron transfer integral. (2) It increases the electron-phonon interaction and the activation energy. Objectively speaking, these variations are in favor of the formation of polarons. As the temperature decreases to 325 K, the polarons delocalization makes it to become “bare” e_*g*_ electrons and the system generates one MIT, exhibiting a PM metallic state. With further decreasing temperature, one PM-FM phase transition emerges at T = 300 K and a long-range FM interaction is established in the system. Therefore, at lower temperature, the ground state of FM metal was observed in this sample.

**Figure 5:**
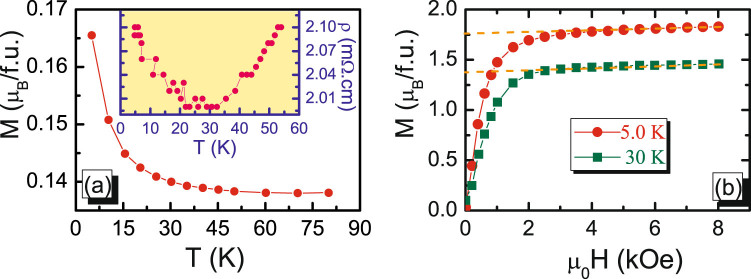
(**a**) Temperature dependence of magnetization at low temperature and inset shows the magnifying plot of resistivity as a function of temperature at low temperature. (**b**) Isothermal magnetization measured at 5.0 and 30 K for La_0.6_Er_0.1_Sr_0.3_MnO_3_.

Apart from the above discussion of the magnetic and electronic transport properties at EPS region, we now turn to the lower temperature region. In fact, as shown in Fig. 1, we have noticed that the magnetization presents a temperature-independence behavior from 300 to 50 K but restarts to increase with decreasing temperature at T < 30 K. The detailed variation can be clearly seen in the magnified plot of Fig. 5(a). Obviously, this feature is related to a new magnetic structure transition. In order to elucidate its physical mechanism, two isothermal magnetization at 5.0 and 30 K are measured. In Fig. 5(b), both magnetizations exhibit a rapid increment and become saturated at 2 kOe. Meanwhile, the spontaneous magnetization (M_*s*_) can be obtained from an extrapolation of the high field curves to H = 0 and the values of M_*S*_ are deduced to be 1.756 and 1.376 *μ*_*B*_, at 5.0 and 30 K respectively. Correspondingly, the change of ΔM_*S*_ is 0.38 *μ*_*B*_. If Er^3+^ ions can form an ordering arrangement on A-sublattice and contribute to ΔM_*S*_, the value should be =0.958 *μ*_*B*_ (The theoretical moment of Er^3+^ ion is 9.58 *μ*_*B*_). The actual change far less than the theoretical value indicates that the increasing magnetization is not due to the ordering magnetic structure of Er^3+^ ions. Here, although the magnetic moment contribution of Er^3+^ ions can’t be excluded completely, we can affirm that the Er^3+^ ions do not form an ordering state on A-site sublattice. This point can be also corroborated from the experimental measurement of electronic transport at the lowest temperature which has been shown in inset of Fig. 5(a). Clearly, at T < 30 K, the resistivity increases with decreasing temperatures. If Er^3+^ ions can be polarized in the lower temperature to form FM ordering which parallels to the external magnetic field, the observed resistivity should reduce rather than raise. On the contrary, if it antiparallels to the external magnetic field, the observed magnetization in the lowest temperature should decrease instead of increase, disagreement with the result of Fig. 5(a). In fact, for the most of perovskite manganite with the magnetic ion doping on A-site sublattice, A-site ordering structure has not been nearly reported. Hence, the large magnetic moments of Er^3+^ ions are definitely not the main contribution for increasing magnetization of Fig. 5(a).

**Figure 6:**
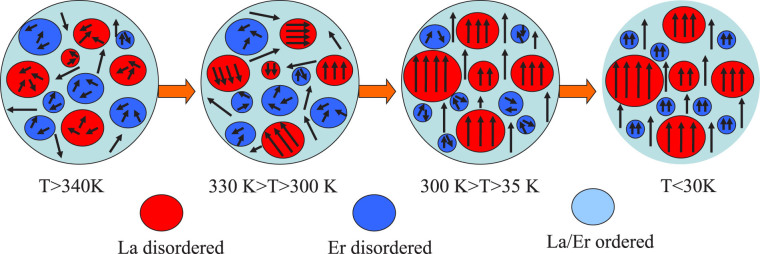
A schematic diagram of the proposed model to describe the magnetic state of La_0.6_Er_0.1_Sr_0.3_MnO_3_ system in different temperature regions.

Based on the above arguments, we can propose a phenomenological model and the following scenario to understand the rise of magnetization and resistivity below 30 K in the LESMO sample. Considering the fact that the Er^3+^ ions randomly occupy A-site sublattice which generates a certain kind of chemical disorder in the system, such an Er^3+^-disorder necessarily induces the presence of La^3+^-disorder since that both of them occupy on A-site sublattice. Here, note that the Er^3+^-doping concentration is only 10% of the entire A-site ions, these disorder regions should be very limited rather than a large-scale. Of course, due to the coexistence of three kinds ions (60%La^3+^, 10%Er^3+^, 30%Sr^2+^) on A-site sublattice, the possibility of Sr^2+^-disorder cannot be excluded as the presence of Er^3+^/La^3+^ disorder. However, the exact determination of the ions distribution on A-site sublattice only depend on the in-depth analysis of superlattice structures in future study. Here, for simplifying the disorder complexity, the Sr^2+^ ions can be thought as an uniform distribution with 30% concentration in all domains. In this case, the whole system can be thought to incorporate three parts, La^3+^-disorder, Er^3+^-disorder and La^3+^/Er^3+^-order. Main part is the domain of La^3+^/Er^3+^-order with the fixed ionic ratio of 6:1. Other parts are a handful of La^3+^- and Er^3+^-disorder microdomain. The La^3+^-disorder domains gather more La^3+^ ions so that its localized concentration is larger than 60%, which is close to the pristine La_0.7_Sr_0.3_MnO_3_ with the high Curie temperature. For Er^3+^-disorder region, the La^3+^ concentration is less than 60% but the Er^3+^ concentration is more than 10% due to more accumulation of Er^3+^ ions. The FM coupling of these regions can be only formed in lower temperature since that the smaller cationic radius of Er^3+^ ions depress Mn-O-Mn bond angles which is mainly responsible for the FM formation of DE interaction. Thus, the whole evolution of magnetic state can be systemically illustrated by Fig. 6. At T > 340 K, all domains can not form a FM coupling so that the sample is in the PM state. As the temperature was decreased below 330 K, the microdomains/clusters of La^3+^-disorder firstly generate some FM interaction but these FM domains are isolated by the region of PM Er^3+^-disorder and La^3+^/Er^3+^-order. Therefore, these microdomains/clusters of La^3+^-disorder are impossible to form a large spontaneous magnetization in the system because the isolated FM clusters can’t generate magnetic correlation among themselves, revealing an intrinsic EPS behavior. With further decreasing temperature below 300 K, the La^3+^/Er^3+^-order region, being a major part of the system, forms an overall PM-FM transition and the systematic magnetic structure becomes a FM state. Under this strong FM background, the isolated FM microdomains of La^3+^-disorder also rotate themselves to make their magnetic moment along with the global FM direction. Meanwhile, besides the FM state formation, the FM regions also grow in size. As a result, the space and domain of Er^3+^-disorder region are suppressed but their moment directions remain random. Such a state rarely change until the molecule thermal energy considerably decreases at T < 30 K. The isolated PM Er^3+^-disorder domains start to form FM coupling and their magnetic moments gradually turn to the orientation of the global FM state. Therefore, an obvious increase of magnetization was observed in the lower temperature(T < 30 K).

Generally, for the electronic transport of perovskite manganites, the increase of systematic FM interaction is helpful for the enhancement of metallic conductivity. However, the sample’s total resistivity is determined by two parts. One is the internal resistivity of domain and the other is the interface resistivity of domain wall. The systematic FM interaction can only decrease the internal resistance of domain but has little or no effect on the interface resistance among neighboring domains. Therefore, at T < 30 K, although the raising magnetization can partly improve the electronic transport in domain, this effect is very limited due to the incremental magnetization incomparable to the entire magnetization. On the contrary, the magnetic transition and the rotation of Er^3+^-disorder domains bring extra strain which changes the structure of domain wall or increases the thickness of domain wall. These influences necessarily lead to a significant enhancement of interface resistance. Consequently, as shown in inset of Fig. 5(a), the total resistance exhibits a continuous rising trend in the lowest temperature.

## Conclusion

In summary, we have studied magnetic and electronic transport properties of the lightly doped perovskite manganite LESMO. The substitution of Er^3+^ for La^3+^ ions reduces the Curie temperature and generates the EPS in this sample due to the induced chemical disorder and extra strain. The observed EPS mainly shows a coexistence of paramagnetic state and ferromagnetic microdomain at high temperature far above T_*C*_. Based on the variation of EPR linewidth Δ*H*_*PP*_ and resistivity, the conductivity in the PM region can well be understood with the hopping of small polarons. With the decrease of temperature, the enhancement of FM microdomain in the size and scope is favor of the delocalization of polarons and triggers the presence of MIT. In the lower temperature of T < 30 K, the FM coupling forms in the microdomains of Er^3+^-disorder and causes an obvious increase of magnetization. However, due to the increase of interface resistance considerably larger than the decrease of internal resistivity of domain, the sample’s total resistance continues to increase below 30 K. Finally, the systematic magnetic state and EPS evolution can be depicted well by a phenomenological model.

## Methods

A polycrystalline LESMO sample was prepared by traditional solid state reaction method. The structure and phase purity of the sample were checked by powder X-ray diffraction (XRD) using Cu K*α* radiation at room temperature. The XRD patterns prove that the sample is pure and a single-phase with orthorhombic structure. The magnetization versus temperature and magnetization versus magnetic field were measured by using a Magnetic Property Measurement System (Quantum Design MPMS 7T-XL) with a superconducting quantum interference device (SQUID) magnetometer. The EPR measurement was performed at selected temperatures using a Bruker EMX-plus model spectrometer with a heater operating at X-band frequencies (*v* ≈ 9.4 GHz). Temperature-dependent electrical resistivity *ρ*(T) was measured using standard four-probe method in a closed cycle refrigerator.
